# Deciphering the Pathological Role of Staphylococcal α-Toxin and Panton–Valentine Leukocidin Using a Novel *Ex Vivo* Human Skin Model

**DOI:** 10.3389/fimmu.2018.00951

**Published:** 2018-05-08

**Authors:** Reuben Olayinka Olaniyi, Laura Pancotto, Luca Grimaldi, Fabio Bagnoli

**Affiliations:** ^1^GSK Srl, Siena, Italy; ^2^Department of Medicine, Surgery and Neuroscience, University of Siena, Siena, Italy

**Keywords:** *Staphylococcus aureus*, alpha-toxin, Panton–Valentine leukocidin, human skin explant model, vaccine

## Abstract

*Staphylococcus aureus* alpha-toxin and Panton–Valentine leukocidin (PVL) have been reported to play critical roles in different animal models of skin infection. These models, however, do not completely recapitulate the human disease due to the host specificity of these toxins as well as the intrinsic anatomical and immunological differences between animals and humans. Human skin explants represent a valid alternative to animal models for studying skin infections. Herein, we developed a human skin explant wound model to study the pathogenic role of alpha-toxin and PVL; inflammatory responses elicited by these toxins; and the neutralizing ability of antibodies to mitigate skin damage. Different concentrations of alpha-toxin and/PVL were applied to superficial wounds on human skin explants. Treatment with alpha-toxin resulted in high tissue toxicity and loss of skin epithelial integrity. PVL induced a milder but significant toxicity with no loss of skin structural integrity. The combination of both toxins resulted in increased tissue toxicity as compared with the individual toxins alone. Treatment of the skin with these toxins also resulted in a decrease of CD45-positive cells in the epidermis. In addition, both toxins induced the release of pro-inflammatory cytokines and chemokines. Finally, antibodies raised against alpha-toxin were able to mitigate tissue toxicity in a concentration-dependent manner. Results from this study confirm the key role of α-toxin in staphylococcal infection of the human skin and suggest a possible cooperation of the two toxins in tissue pathology.

## Introduction

*Staphylococcus aureus* is an important human pathogen responsible for a vast array of diseases such as skin and soft tissue infection (SSTI), bacteremia, necrotizing pneumonia, infective endocarditis, and osteomyelitis. SSTI is the most frequent disease associated with *S. aureus*. The emergence of methicillin-resistant *S. aureus* has made treatment increasingly difficult, and there is currently no vaccine available on the market for its prevention (1, 2).

The success of *S. aureus* as a pathogen can be attributed to the large armamentarium of viru-lence factors it harbors. The role of several of these factors in SSTI pathogenesis has been widely studied (3). β-Barrel pore-forming toxins have been linked to SSTI, of these, α-toxin (Hla) is unarguably one of the most studied (4, 5). Its role in skin infections has been well elucidated in both *in vitro* and in animal models (3, 6). The contribution of Hla to SSTI in humans is, however, not fully elucidated (7). In addition to Hla, epidemiological data suggest a link between the expressions of Panton–Valentine leukocidin (PVL) to severe cases of community-acquired SSTI. Nonetheless, the role of PVL in human infection remains to be clarified (8, 9).

Identification of receptors for these toxins has provided insights into the host and tissue specificity of Hla and PVL. ADAM10, a metalloprotease and disintegrin, was identified as the cellular receptor for Hla. It is highly expressed by endothelial, epithelial, and several immune cells. The lack of expression or reduction in expression of ADAM10 has been linked to reduced susceptibility to Hla toxicity in mice (4). PVL targets chemokine-specific complement and cytokine receptors expressed on neutrophils and other innate cells such as macrophages. Host susceptibility to PVL has been linked to the diversity of the C5aR receptor among different species. Recent studies using a humanized mouse model showed an increased susceptibility to PVL mediated pathology in the presence of human immune cells (10, 11). This observation accentuates the relevance of using clinically relevant models in *S. aureus* research (12). The use of animal models for staphylococcal research, such as the ones based on mice, have been limited due to host-specific virulence factors released by *S. aureus* (5) and to the anatomical/immunological differences with humans (13). Monolayers of keratinocytes (2D) have been widely used to study *S. aureus* host–pathogen interaction (14–16). More recently, organotypic three-dimensional (3D) skin cultures have been adopted in staphylococcal research (13, 17–19). While these *in vitro* models have undoubtedly been useful for studying interactions of the pathogen with the epithelial components of the skin, they do not recapitulate the complex interaction occurring *in vivo*.

In this study, we developed a human skin explant model with the aim of studying the interactions of *S. aureus* within the complex environment of human skin. The model was used to characterize the pathologic effect of Hla and PVL, elucidate the inflammatory responses elicited by these toxins as well as assess the inhibitory activity of toxin-specific antibodies.

## Materials and Methods

### *Ex Vivo* Skin Preparation

Human skin explants were received as surgical waste from healthy patients undergoing abdominoplasty. Samples were transported in culture medium [DMEM (Life Technologies) supplemented with 10% fetal bovine serums (Life Technologies) and 1× antibiotic–antimycotic (15240062, Life Technologies)] and processed within 3 h of collection. Skin explants were washed three times in culture medium and 1× phosphate-buffered saline (PBS). To generate a superficial wound for toxin stimulation and infection, the *stratum corneum* was stripped 20–30 times using an adhesive tape. 8-mm (in diameter) biopsies were taken using disposable surgical biopsy punches (code: BP-80F, Kai Medical). Skin punches were placed in 12 mm insert wells with 0.4 µm pore size membrane (code: CLS3460, Sigma-Aldrich). No more than one skin punch was place in a 12 mm insert. The inserts were placed in 12-well plates prefilled with 1 ml of culture medium. Explants were cultured at air–liquid interface (Figure S1 in Supplementary Material) at 37°C and 5% CO_2_. For infection experiments, skin explant culture medium was supplemented with 10 µg/ml of chloramphenicol without fetal bovine serum.

### PrestoBlue Assay

Skin explant viability was assessed using PrestoBlue (a resazurin-based reagent) viability reagent (Invitrogen Corporation, Carlsbad, CA, USA) following the manufacturer’s recommendations. 8 mm skin biopsies were cultured *ex vivo* for a week in tissue culture medium. After 0, 24, and 168 h in culture, skin biopsies were further incubated in 300 µl of 10% PrestoBlue (in DMEM with antibiotics) for 3 h at 37°C, and 5% CO_2_. Fluorescence emission was measured at 560 nm excitation and 590 nm emission wavelengths.

### Toxin Stimulation

Equal molar concentrations of recombinant LukS-PV tag-free (code: 0540-001, IBT Bioservices) and recombinant LukF-PV tag-free (code: 0540-002, IBT Bioservices) were premixed to generate a working concentration of PVL. α-toxin (Hla) and its detoxified mutant Hla_H35L_ were produced as previously described (20). Lipopolysaccharide contamination in purified proteins was assessed using the LAL test (from Charles River). Endotoxin content was less than 0.05 EU per microgram of purified protein. Skin explants were stimulated within 2 h after being placed in culture with PVL or Hla at a concentration of 1 and 5 µg per 8 mm (diameter) skin biopsy for 24 and 72 h. For experiments where the two toxins were combined, α-toxin and PVL at 1 µg each (1:1 M concentration of LukF-PV and LukS-PV) were applied on human skin. Hla_H35L_ and LukS-PV were used as negative controls. Spent media were stored at −80°C for further analyses.

### Bacterial Strains, Growth Conditions, and Skin Infection

*Staphylococcus aureus* strain USA300 LAC wild type, its isogenic *Δhla* mutant (hla:ermB) and *hla* complemented (hla:ermB-phla) strains were used for infection experiments. Mutants and complemented strains were generated as previously described (21). All strains contain pOS1 plasmid encoding a gene that confers chloramphenicol resistance. Frozen stocks of bacteria were streaked onto fresh tryptic soy agar (TSA) supplemented with chloramphenicol (10 µg/ml) and incubated overnight at 37°C. A single colony was inoculated in a fresh tryptic soy broth containing chloramphenicol (10 µg/ml) and incubated overnight with shaking (250 rpm) at 37°C to obtain bacterial cells at the stationary phase of growth. Cells were harvested by centrifugation at 4,000 rpm for 10 min, washed and resuspended in PBS. Skin explants were kept in culture for 16–18 h before infection to ensure adequate diffusion of chloramphenicol. 1 µl of bacterial suspension corresponding to 5 × 10^5^ colony-forming units (CFUs) was applied to the surface of stripped skin explants. Infected skin explants were incubated for 24 and 72 h at 37°C and 5% CO_2_. The spent medium was stored at −80°C for further analysis.

### Recovery of *S. aureus* From Human Skin Explants

Bacteria were recovered from skin using an already described protocol (22). Briefly, *S. aureus* was dislodged from unwashed infected skin explants by vortex mixing for 2 min in 250 µl sterile PBS. Supernatants were serially diluted in PBS and spread on TSA containing 5% sheep’s blood and 10 µg/ml of chloramphenicol.

### Lactate Dehydrogenase (LDH) Assay

Toxin-induced toxicity was determined by measuring the level of LDH released into the culture medium by cells of the skin. LDH activity in the culture spent medium was assayed using a CytoTox 96 kit (Promega Corporation). 50 µl reconstituted substrate mix was added to 50 µl of diluted tissue spent medium (1:50). The solution was incubated at room temperature for 30 min. The reaction was stopped by adding stop solution provided in the kit, and the absorbance measured at 490 nm with a TECAN spectrophotometer.

### Cytokine Analysis

Inflammatory cytokines [interferon-gamma (IFN-γ), IL-1β, IL-2, IL-4, IL-6, IL-8, IL-10, interleukin 12p70 (IL-12p70), IL-13, and tumor necrosis factor alpha (TNF-α)] and chemokines [Eotaxin, Eotaxin-3, IL-8, interferon-gamma-induced protein 10 (IP-10), monocyte chemoattractant protein 1 (MCP-1), MCP-4, macrophage-derived chemokine (MDC), MIP-1α, MIP-1β, and TARC] released into tissue spent medium were measured using MesoScale Discovery technology (Rockville), using a V-Plex pro-inflammatory/chemokine panel 1 human kits following manufacturer’s recommendations.

### Purification of Total Immunoglobulin

Total immunoglobulin G (IgG) was isolated from sera of rabbits immunized with Hla_H35L_ or Alum (Adjuvant) using Ab spinTrap columns (GE Healthcare Lifesciences, code: 28-4083-47) following the manufacturer’s recommendation.

### Histological Analysis and Immunostaining

Skin tissues were placed in optimum cutting temperature compound (Sakura Finetek), then snap frozen with ethanol-dry ice and stored at −80°C until needed for sectioning. Cryosections of 8 µm were obtained using a Leica cryostat CM1950 and fixed for 10 min in 4% formaldehyde at room temperature. Fixed samples were either stained immediately or dehydrated in absolute ethanol for 2 min. Dehydrated cryosections were stored at −20°C until needed for staining. Hematoxylin and eosin (H&E) staining were performed on 8 µm skin cryosections with Leica ST5020 Multistainer and images acquired with Leica optical microscope. Immunofluorescence staining was performed with mouse anti-E-Cadherin (clone 36/E-Cadherin, BD Transduction Laboratories), mouse anti-CD45 (LCA) BioLegend, rabbit anti-ADAM10 (ab1997, Abcam), goat anti-rabbit Alexa Fluor 568 (10 µg/ml code: A-11011, Life technologies), and goat anti-mouse Alexa Fluor 568 (10 µg/ml, code: A-21124, Life Technologies). Staining was visualized using a Zeiss LSM700 confocal microscope. All image processing and analyses were performed with ImageJ software.

### TUNEL Assay

Apoptosis was assessed on skin cryosections using an *In Situ* Cell Death Detection Kit, Fluorescein (Roche 11684795910, Roche Diagnostics GmbH Roche Applied Science Mannheim, Germany) according to the manufacturer’s protocol.

### Ethical Statements

Human skin samples were received as surgical waste from healthy patients undergoing abdominoplasty with full ethical approval from Azienda Ospedaliera Universitaria Senese and informed patient consent.

### Statistical Analysis

The data were analyzed using the GraphPad Prism v.7.0 software Statistical analysis was performed using one-way or two-way ANOVA and Tukey’s multiple comparison tests. Differences were considered significant at *p* ≤ 0.05.

## Results

### Skin Explant Viability and Model Setup

**Figure 1:**
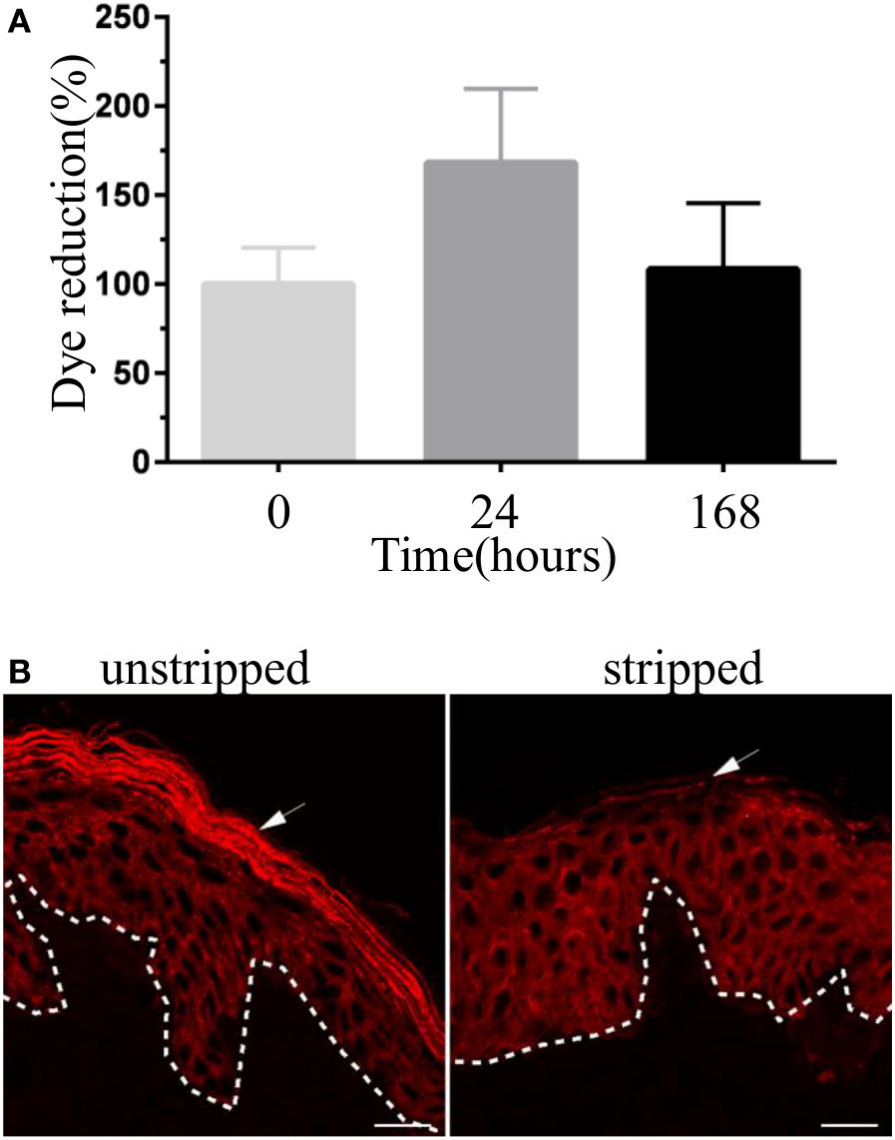
Validation of human skin explant model. **(A)** The viability of human skin explant in culture. Each bar represents the reduction of PrestoBlue dye staining (a cell viability testing reagent) by skin samples at different time points in culture compared with freshly received skin tissue (time 0). Results are pooled from three independent experiments. **(B)** Immunofluorescence images showing skin cryosections of unstripped and stripped human skin tissue stained with anti-pan-cytokeratin antibody. Stripped skin lacks stratum corneum (arrow). Scale bar: 20 µm.

To verify the viability of human skin tissue during culture (see Figure S1 in Supplementary Material), we assessed the ability of skin cells to reduce PrestoBlue dye, a resazurin-based cell viability testing reagent. Dye reduction was measured for up to 7 days (Figure 1A). Results indicate that human skin explant remained viable over the culture period. Of note, this assay does not provide an absolute count of viable cells in tissues. An increase in dye reduction is, however, consistent with the metabolic activity of tissue cells, and this correlates with the numbers of viable cells in the tissue explants. For infection and treatment with purified toxins, human skin explants were tape stripped to remove the *stratum corneum*. Indeed this layer of the skin provides resistance against infection and toxin penetration (data not shown). Staining with pan-cytokeratin antibodies revealed the complete absence of *stratum corneum* from the stripped explants (Figure 1B).

### *S. aureus* α-toxin and PVL Mediate Tissue Toxicity

**Figure 2:**
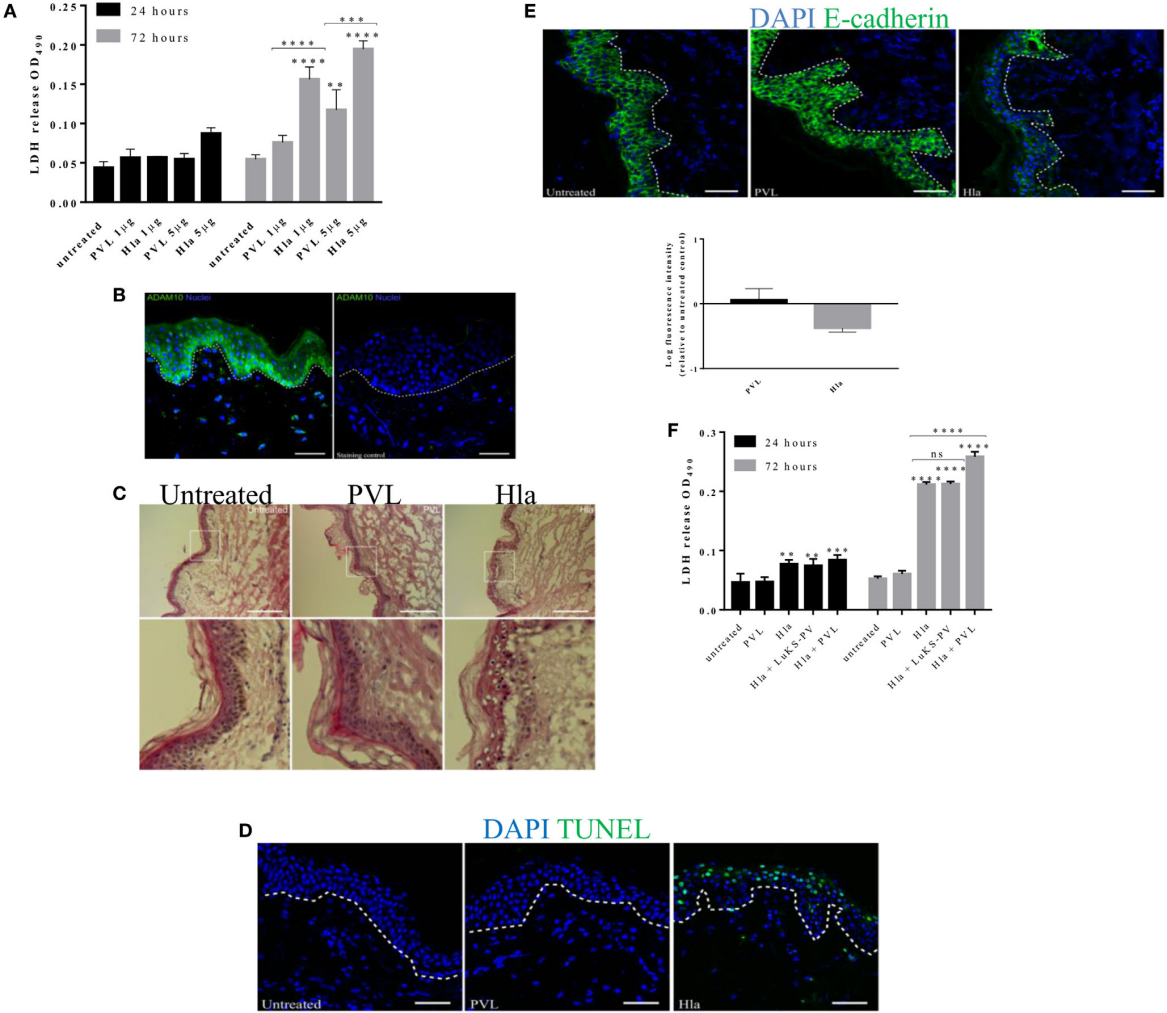
The role of Hla and Panton–Valentine leukocidin (PVL) in tissue toxicity and disruption of skin integrity. Human skin explants were treated with 1 and 5 µg of α-toxin or PVL and a cocktail of both toxins at 1 µg each. **(A)** Lactate dehydrogenase (LDH) released from human skin during 24–72 h of treatment with staphylococcal toxins. Data are combined results from three independent experiments Each bar represents the mean ± SD, *N* ≥ 2 for each experiment. Where *N* ≥ 2 represents intra-experimental replicates. **(B)** Immunofluorescence image of human skin section showing the expression of ADAM10 within the layers the epidermis. Scale bar 50 µm. **(C)** Hematoxylin and eosin staining of skin cryosections taken 24 h post toxin treatment. White box represents a magnified area of the image. Scale bar is 100 µm. **(D)** Cryosections of human skin explant showing TUNEL-positive cells (in green) after 24 h treatment with Hla or PVL. Scale bar 50 µm. **(E)** Immunofluorescence images of E-cadherin signal on skin cryosections 24 h post toxin stimulation. Scale bar 50 µm. **(F)** LDH released from human skin during 24 and 72 h of treatment with a cocktail of Hla and PVL at 1 µg each. Each bar represents the mean ± SD (*N* = 3). Statistical analysis was performed by two-way ANOVA, with Tukey’s multiple comparison tests, **p ≤ 0.001, ***p ≤ 0.0002, and ****p < 0.0001. Asterisks on bars are a comparison between the treated and untreated skin while lines across bars are comparison between groups. White dotted lines in images represent the boundary between epidermis and dermis.

To elucidate the role of *S. aureus* toxins in the pathology of skin tissue, stripped skin explants were treated with 1–5 µg of Hla and or PVL (1:1 equal molar concentrations of LukF-PV and LukS-PV). Tissue damage was assessed by measuring: the level of LDH in tissue spent medium, the number of apoptotic cells within tissue cryosections and H&E staining for histopathological changes. Amount of LDH in the medium of skin treated with Hla significantly increased at 24 h post-treatment and increased further after 72 h in a concentration-dependent manner. TUNEL assay on skin sections 24 h post-treatment also revealed several apoptotic cells within the epidermis (see Figure S2 in Supplementary Material). PVL treatment did not result in increased LDH release at an earlier time point while a significant increase was observed 72 h post-treatment at 5 µg (Figure 2A). LukS-PV (a single subunit of PVL) and detoxified Hla (Hla_H35L_) were used as negative controls for active PVL and Hla toxins, respectively. The level of LDH in the spent medium of the skin treated with either negative controls was similar to those obtained in the untreated. Hla-mediated toxicity has been shown to depend on ADAM10 expression (23). Immunofluorescence images of skin sections revealed an intense staining of ADAM10 within keratinocytes of the *stratum basale* in comparison with keratinocytes already committed to differentiation (Figure 2B). Histological analysis revealed a significant loss of skin epithelial integrity in skin treated with Hla at 24 h which increased after 72 h (data not shown). PVL treatment of the skin did not result in any observable loss of epithelial integrity in all tested concentrations and time points (Figure 2C). Analysis of tissue cryosections at 24 h showed TUNEL-positive cells in the epidermis of Hla-treated skin biopsy at all tested concentrations while none was observed in PVL treated skin (Figure 2D). Assessment of E-cadherin expression, an adherence junction protein, revealed a reduction in the fluorescence intensity of this protein in explants treated with Hla but not PVL (Figure 2E). Treatment with PVL was found to increase E-cadherin levels (Figure 2E). PVL, at low concentration (1 µg), did not induce a significant increase in LDH or in observable skin pathology in this study (Figure 2A). We wondered if PVL may cooperate with Hla in tissue damage. We therefore tested the effect of the two toxins combined. Treatment of the skin with a cocktail of 1 µg of both toxins resulted in a higher LDH release at 72 h when compared with individual toxins, thus suggesting a potential cooperative effect (Figure 2F). Increasing the concentration of PVL above 1 µg did not result in a significant increase in LDH release (see Figure S3 in Supplementary Material). The observed increase in cytolysis depends on both components of PVL as a cocktail of Hla and a subunit of PVL (LukS-PV) did not result in increased LDH release. Taken together, these findings confirm the role of Hla in human skin infection and suggest a possible interplay of the two toxins in tissue pathology.

### α-toxin and PVL Mediate the Loss of CD45-Positive Cells From Human Skin

**Figure 3:**
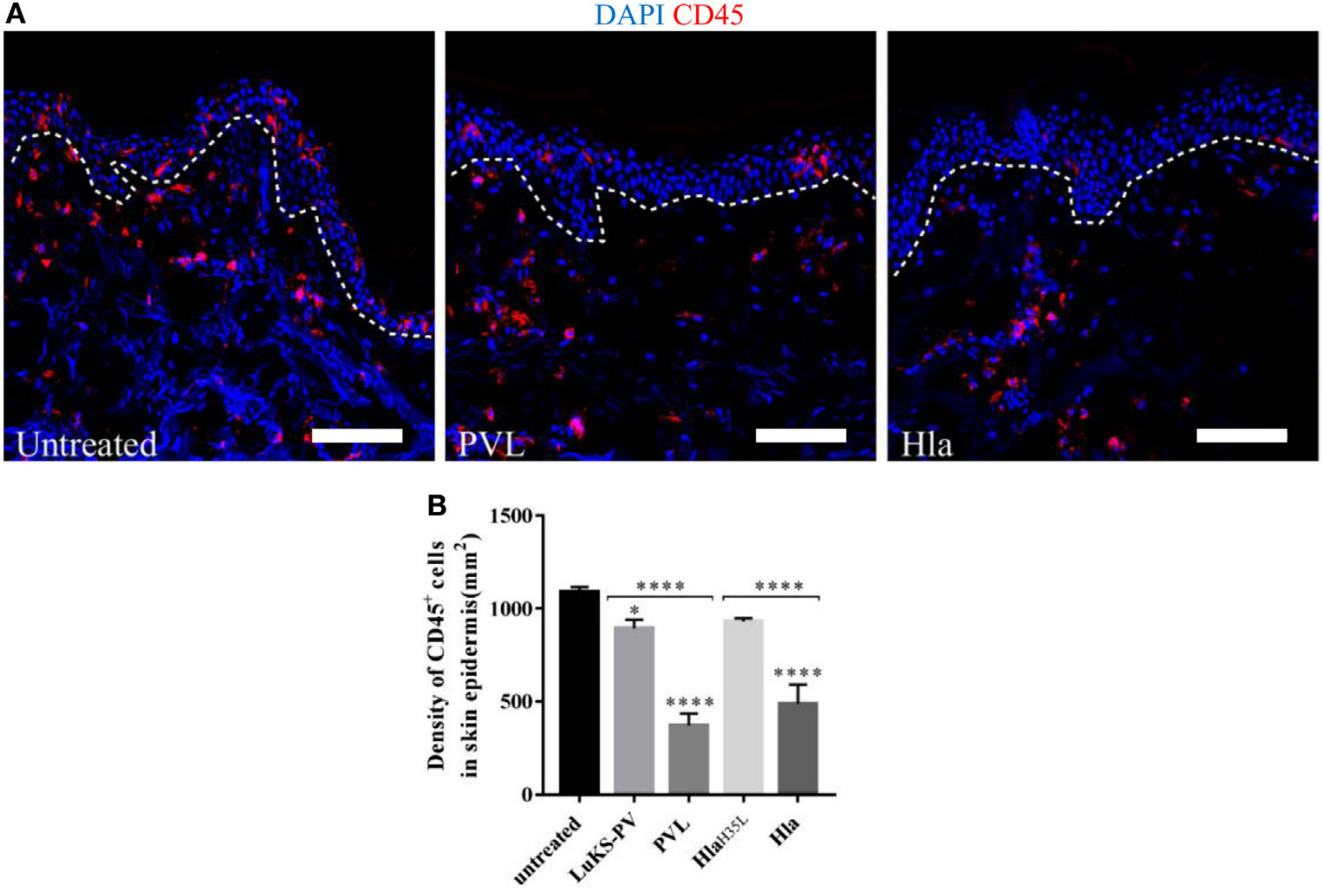
**(A)** Cryosections of human skin explants showing the distribution of CD45-positive cells in the epidermis of human skin 24 h post-treatment with 5 µg Hla or Panton–Valentine leukocidin (PVL). Scale bar is 100 μm. The white dotted line represents the border between the epidermis and dermis. **(B)** The density of CD45 cells was calculated by counting CD45-positive cells in three to four random fields of skin sections. The density of cells measured as cell-density per square micrometers but expressed in cell-density per square millimeters. Statistically significant differences were determined by one-way ANOVA, with Tukey’s multiple comparison tests, *p < 0.05 and ****p ≤ 0.0001. Asterisks on bars are comparisons between untreated and treated groups while lines above bars are comparisons between the treated groups.

Staphylococcal Hla and PVL have been observed in animal models of infection to target a broad range of immune cells thereby compromising host response to infection (4, 7, 12, 24). To assess the impact of these toxins on skin resident immune cells, wounded human skin explants were treated with 5 µg of either Hla or PVL. Of note, 5 µg was the concentration of toxins that resulted in highest LDH release under our experimental conditions (see Figure 2A). The loss of skin resident immune cells was assessed by confocal microscopy using a CD45 antibody (pan-leukocyte antibody). Immunofluorescence images revealed a marked reduction of CD45-positive cells in the epidermis of skin treated with Hla or PVL as compared with the untreated samples (Figure 3A). Counting the number of CD45-positive cells in three to four random fields of tissue sections showed a significant reduction in the density of these cells in Hla and PVL treated skin as compared with untreated skin sample (Figure 3B). The reduction of this cell type from the epidermis is likely due to toxicity effect as a mutated form of Hla (Hla_H35L_) and a single subunit of PVL (LukS-PV) resulted in minimal reduction in the density of CD45 cells in the epidermis. TUNEL assay on these skin sections revealed apoptotic CD45 cells within the dermis of skin treated with Hla while little to none was observed in PVL and untreated group (see Figure S4 in Supplementary Material).

### α-toxin and PVL Induce Inflammatory Response in Human Skin

**Figure 4:**
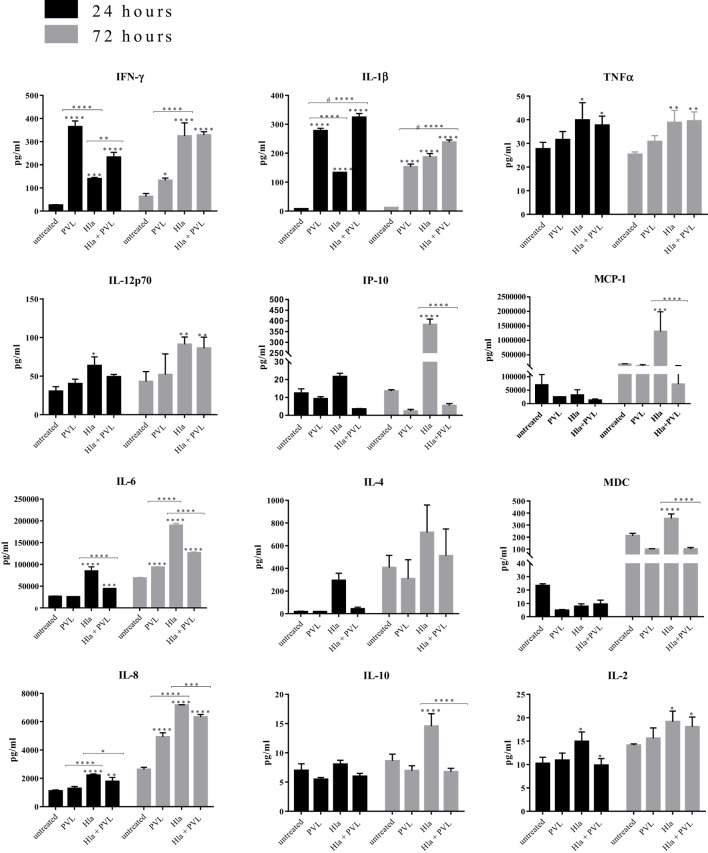
Hla and Panton–Valentine leukocidin (PVL) elicit the release of pro-inflammatory mediators in human skin. Human skin explants were treated with 1 µg of Hla, PVL, or a cocktail of both toxins at 1 µg each. Each bar represents the mean ± SD of three analyses from the skin of one donor. In addition, results were confirmed using skin explants from two additional donors in independent experiments (data not shown). Statistically significant differences were determined by two-way ANOVA, with Tukey’s multiple comparison tests, *p < 0.05, **p ≤ 0.001, ***p ≤ 0.0002, and ****p < 0.0001. Asterisks on bars are comparisons between untreated and treated groups while lines above bars are comparisons between groups. ^#^Represents comparisons between PVL and Hla + PVL.

To elucidate if in addition to cell toxicity the two toxins also induce inflammatory responses in skin resident cells, the spent culture medium of toxin-treated skin explants were analyzed for the presence of inflammatory mediators using a Mesoscale Discovery V-plex pro-inflammatory and chemokine kits. Results at both 24 and 72 h post-treatment, indicates that Hla significantly induced Th1 mediators: IFN-γ, TNF-α, IL-12p70, IL-2, MCP-1, and IP-10. PVL induced a significant increase of Th1 mediator IFN-γ (Figure 4). In addition, Hla induced Th2 mediators IL-4, IL-10, and MDC, while IL-1β and IL-6, which can promote differentiation of T-helper cells toward Th17 (25, 26) were significantly induced by both toxins. IL-8, a neutrophil chemotactic factor, was significantly induced by both toxins. With the exception of IFN-γ and IL-1 β, Hla and PVL induced a similar response pattern at all tested concentrations (see Figure S5 in Supplementary Material). Treatment with Hla and PVL cocktail resulted in a significant reduction of most inflammatory cytokines/chemokine (particularly at the earlier time point) except for IL-1β in which showed a significant increase when compared with the individual toxins (Figure 4).

### α-toxin Contributes to *S. aureus*-Mediated Tissue Toxicity

**Figure 5:**
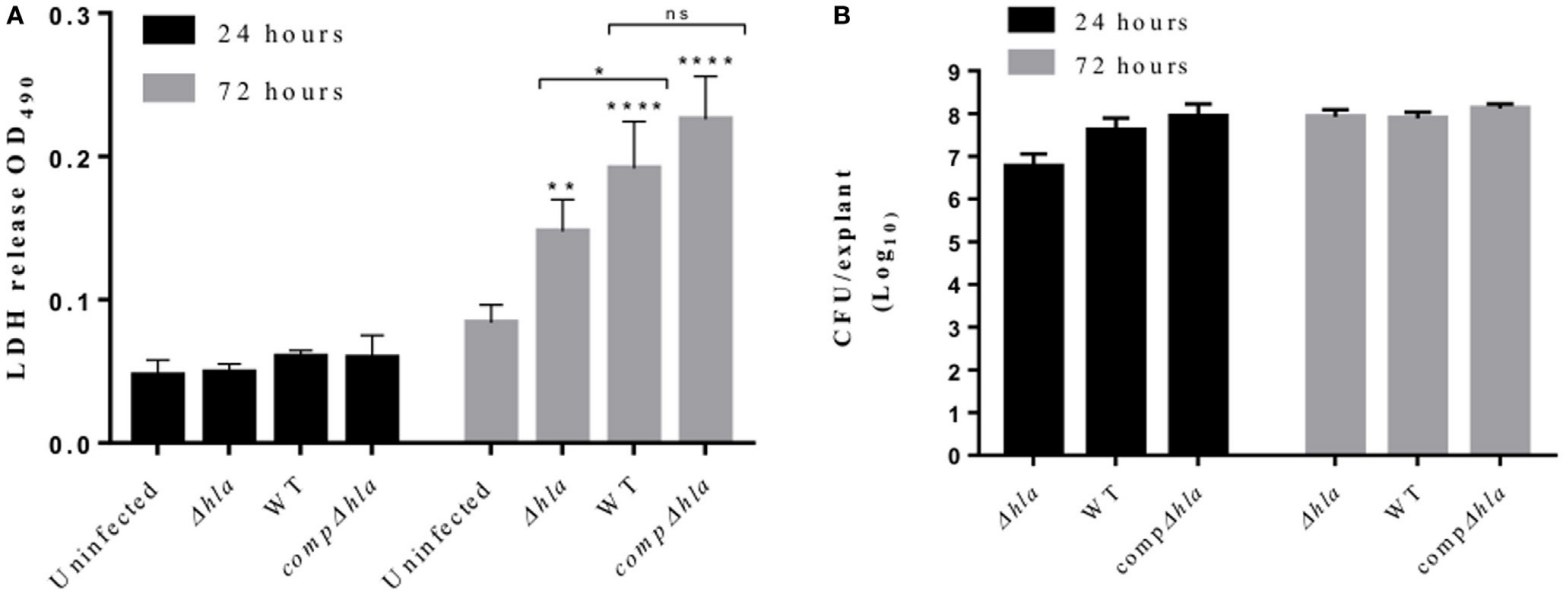
Hla mediates tissue toxicity during *Staphylococcus aureus* skin infection. Human skin explants were infected with *S. aureus* USA300 LAC wild type, its isogenic *hla* mutant, and the complemented strain (comp*Δhla*). **(A)** Lactate dehydrogenase (LDH) released from the skin during infection with *S. aureus* wild-type and *hla* mutant LAC USA300 strains. Each bar represents the mean ± SD (*N* = 3). **(B)** Colony-forming unit (CFU) of *S. aureus* recovered 24 and 72 h post-infection. Each bar represents the mean ± SD (*N* = 3). Statistically significant differences were determined by two-way ANOVA, with Tukey’s multiple comparison tests, *p < 0.05, **p ≤ 0.001, and ****p < 0.0001. Asterisks on bars are comparisons between infected and uninfected groups while lines are the comparison between infected groups.

To understand if our observations using purified Hla truly reflect toxin activity during infection, wounded human skin explants were infected with 5 × 10^5^ CFUs of wild-type *S. aureus* USA300 LAC or its isogenic mutant (hla:ermB). Tissue pathology was assessed with LDH assay. As shown in Figure 5A, both wild-type *S. aureus* and its isogenic mutant (*Δhla*) were able to induce tissue toxicity. The wild-type strain, however, induced significantly higher tissue damage as compared with the mutant strain. Complementation of the mutant strain with *hla* (hla:ermB-phla) restored toxicity to wild-type level (Figure 5A). Of note, there was no significant difference in the CFU recovered at any time points (Figure 5B), thus suggesting that Hla production does not enhance bacterial survival on human skin.

### α-toxin Antibodies Mitigate Tissue Damage but Do Not Interfere With Bacterial Growth

**Figure 6:**
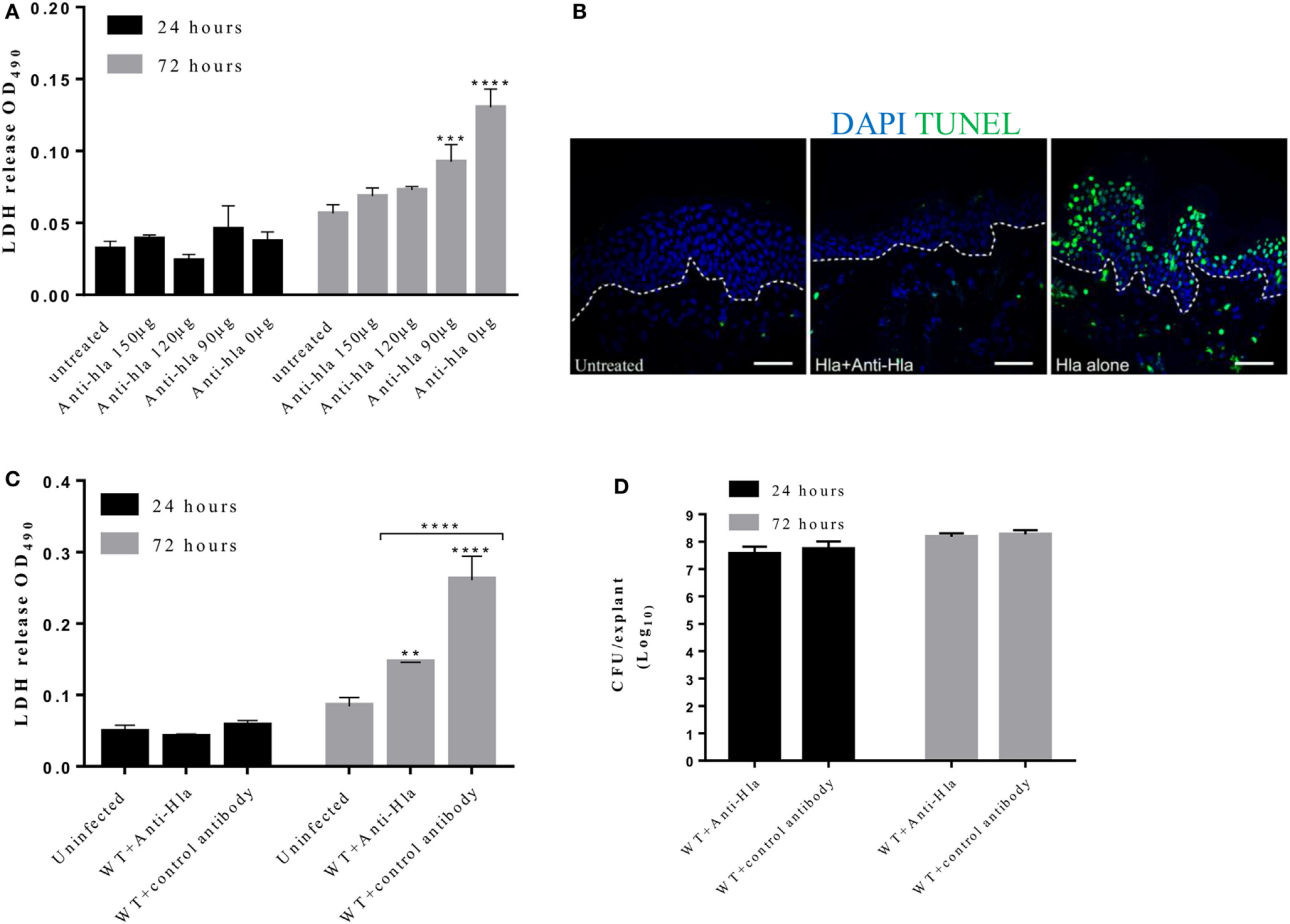
Anti-Hla antibodies mitigate tissue cytolysis but not bacteria survival during infection. Human skin explants were treated with 5 µg of Hla in combination with increasing concentrations of rabbit anti-Hla antibodies. For infection experiments, skin explants were infected with 5 × 10^5^ colony-forming unit (CFU) of *Staphylococcus aureus* USA300 wild type in combination with 150 µg of purified rabbit anti-Hla antibodies or unspecific antibodies. **(A)** Inhibition of purified Hla activity by anti-Hla antibodies in a concentration-dependent manner. Each bar represents the mean ± SD (*N* = 3). **(B)** Immunofluorescence images of skin sections showing TUNEL-positive cells in the skin treated with Hla in combination with 150 µg of anti-Hla antibodies. White dotted lines represent the boundary between the epidermis and dermis. Scale bar is 50 µm. **(C)** Lactate dehydrogenase (LDH) content in the medium of infected skin. Each bar represents the mean ± SD (*N* = 3). **(D)** Bacteria CFU recovered from the skin 24 and 72 h post-infection. Each bar represents the mean ± SD (*N* = 3). Statistically significant differences were determined by two-way ANOVA, with Tukey’s multiple comparison tests, **p ≤ 0.05, ***p ≤ 0.0002, and ****p < 0.0001.

Studies in mouse models have demonstrated the role of Hla antibodies in mitigating severe staphylococcal skin diseases (27, 28). To determine whether Hla antibodies can decrease the damage mediated by the toxin to human skin, a dose–response experiment was performed in which skin explants were treated with 5 µg of Hla together with increasing concentration of Hla antibodies (total IgG purified from rabbits immunized with Hla_H35L_). The antibody neutralizing effect on Hla-induced toxicity was measured using the LDH and TUNEL assay. Results showed that antibodies were able to block Hla-mediated toxicity and damage of tissue epithelial integrity in an antibody concentration-dependent manner (Figure 6A). Skin explants, treated with both the toxin and toxin-specific antibodies, showed a marked reduction in LDH content in the spent medium. Reduction of TUNEL-positive cells was also observed at 24 h post-treatment in skin treated with Hla together with antibodies (Figure 6B).

We also assessed the ability of these antibodies to neutralize Hla activity during skin infection. Skin explants were infected with 5 × 10^5^ CFU *S. aureus* USA300 LAC with or without 150 µg of Hla antibodies. Explants that received Hla antibodies showed a significant reduction in toxicity as compared with the skin treated with unspecific antibodies (Figure 6C). However, the antibodies had no effect on the ability of *S. aureus* to grow and infect human skin as there was no significant difference in the recovered CFU at all time points (Figure 6D).

## Discussion

*Staphylococcus aureus* is an important human pathogen that accounts for the majority of SSTI. The host specificity of staphylococcal virulence factors makes it difficult to translate findings in animal models to humans. The use of isolated cells or 3D cell cultures does not fully recapitulate the complex interaction between *S. aureus* and human tissues. Furthermore, several pieces of evidence from animal models suggest *S. aureus* pathogenesis varies between tissues (29, 30). Recognizing the limitations of some of the models currently used for *S. aureus* research, we developed an *ex vivo* human skin explant model to study staphylococcal virulent factors, elucidate immune responses of skin cells against these factors, and evaluate the efficacy of antibodies in blocking their activity. The *stratum corneum* of the epidermis was removed by tape stripping for mimicking superficial wounds which are thought to represent the first event in many SSTI (31).

Using this model, we showed that both purified Hla and PVL were able to induce tissue toxicity in a concentration and time-dependent manner. Hla induced toxicity and disruption of skin epithelial integrity at all tested concentrations. Significant cytolytic activity of Hla in human skin seems to occur at a later time point during treatment. A recent study on human 3D lung model (32) showed an increase in toxicity of lung cells within 24 h of treatment at a concentration lower than what we used. This variation in susceptibility between the skin and lung cells may be ascribed to the level of ADAM10 expression in these tissues (33). While Mairpady et al. (32) showed a high and uniform distribution of ADAM10 in stratified lung epithelial cells, our confocal microscopy results showed a differential expression of this receptor in the *strata* of the skin. We observed a reduced ADAM10 signal in cells of the *stratum spinosum* and *granulosum* as compared with undifferentiated cells of *stratum basale* that stained strongly with ADAM10 antibodies. Our observation is in line with human tissue proteomic data showing a higher expression of ADAM10 in lung tissue as compared with skin (33). Studies have shown that Hla activation of ADAM10 results in ADAM10-dependent E-cadherin cleavage and subsequent disruption of epithelial integrity (23). Our findings revealed that Hla treatment was able to induce the cleavage of E-cadherin at early time points and at all tested concentrations. This may facilitate the invasion of *S. aureus* during infection. Infecting the human skin with the wild type and *hla* mutant strains confirms observations done in animal skin infection models. Indeed, although this toxin significantly increased the tissue damage, it showed no significant contribution to bacterial growth or survival as there was no significant difference in the CFU recovered at any time points after infection. Although there are epidemiological data linking the expression of PVL to disease severity in human (34, 35), experimental data from animal models remain controversial. Therefore, we decided to also evaluate the role of this toxin in human skin pathology. Our results indicate that PVL is able to induce a mild but significant toxicity at the highest tested concentration in human skin. Hla showed higher tissue toxicity as compared with PVL. The different level of toxicity between Hla and PVL could be attributable to cell susceptibility. While Hla is known to target several cells (both epithelial and immune cells), PVL targets mainly neutrophils and to a lesser extent Langerhans cells, dendritic cells, and macrophages. The low susceptibility of Langerhans and dendritic cells, as well as macrophages to PVL killing (22, 31), would explain why a high concentration of the toxin is needed to see a significant increase in toxicity in the absence of recruited neutrophils (missing in the skin explants). Our data also showed that combining both PVL and Hla resulted in increased tissue toxicity as compared with the individual toxins. These data are in agreement with a recently published work where a combination of Hla and PVL was shown to increase lung tissue damage (32). This cooperative effect requires active toxins since a combination of a subunit of PVL (LukS-PV) with Hla or the use of Hla_H35L_ did not result in increased toxicity.

Human skin contains several permanent immune cells that play crucial roles in cutaneous immune response (36). Langerhans cells resident in the epidermis are one of the first responders during an assault to the skin. Our data revealed a reduction in the number of cells positive for the pan-leukocyte marker CD45 in the epidermis of the skin treated with either Hla or PVL at 24 h post-treatment. The loss of these cells is not due to spontaneous migration from the skin as treatment with a mutated form of Hla (Hla_H35L_) and a subunit of PVL (LukS-PV) did not result in a significant reduction in the density of CD45-positive cells in the epidermis as compared with the untreated skin. The loss of skin resident immune cells induced by these toxins may dampen a robust immune response during staphylococcal infection. Indeed, previous studies have linked the expression of these toxins to dampened immune response in different infection models (24, 37).

The cytokine analysis conducted herein shows that Hla induced a mixed response. Th1 and Th2 mediators as well as IL-1β and IL-6, which can promote differentiation of T-helper cells toward Th17 were detected in the medium of Hla-treated skin tissue. Previous study by Nygaard et al. showed that Hla induced a mixed response from human immune cells (38). The combination of these cytokines/chemokines induced by Hla may enhance disease pathogenesis by causing excessive inflammation or modulating the immune response to create a favorable environment for *S. aureus* during infection (39). Some of the cytokines in the medium of Hla-treated skin seemed to increase at 72 h in comparison with a later time point. This increase may be due to greater cell lyses observed at this time point. PVL treatment resulted mainly in the secretion of Th1 mediators IL-1β and IL-6, which can promote differentiation of T-helper cells toward Th17. In line with previous studies (4, 32, 40), we observed increased induction of the neutrophil chemotactic factor IL-8 by both toxins. Pro-inflammatory and immune-activating properties of PVL at low concentrations have been previously reported by Yoong and Pier in a mouse model of pneumonia infection (41). With the exception of IL-1β, treating the skin with a cocktail of both Hla and PVL resulted in the secretion of lower amount of cytokines/chemokines when compared with the amount secreted following treatment with Hla alone. The increase of IL-1β may be due to pyroptosis, a caspase 1-dependent form of inflammatory cell death, induced by the toxin treatment. This form of cell death is accompanied by increased accumulation of cytokines (mostly IL-1β). Studies in animal models have demonstrated that antibodies against key *S. aureus* toxins are efficacious in reducing disease severity during infection (42–44). Corroborating these studies, our data showed that Hla-specific antibodies were able to protect human skin from structural damage and reduce tissue toxicity due to Hla activity. The antibodies were also able to decrease tissue toxicity due to infection with live bacteria. However, Hla antibodies had no effect on bacterial counts. Our finding further underscores the importance of targeting Hla through immunotherapy or active vaccination for coping with *S. aureus* skin infections.

In conclusion, we showed that human skin explant represents a clinically relevant model for *S. aureus* research. We believe that this model can help to better understand tissue-specific patho-logy induced by staphylococcal virulence factors. Although this model recapitulates the complex composition and interaction of skin resident cells, it lacks the recruitment of neutrophils and other immune cells occurring in response to an infection. Neutrophils are essential for disease clearance and are major targets of *S. aureus* exotoxins. Recent evidence from a humanized mouse model has shown the relevance of neutrophils in PVL induced pathology (10, 11). In addition, the low number of skin samples from different donors tested is another limitation of this current study. Notwithstanding, this model is relevant in that it can help us to understand the early pathophysiology and inflammatory responses to staphylococcal infection that eventually induce recruitment of neutrophils and other immune cells, ultimately leading to bacterial containment or clearance.

## Ethics Statement

Human skin samples were received as surgical waste from healthy patients undergoing abdominoplasty with full ethical approval from Azienda Ospedaliera Universitaria Senese and informed patient consent.

## Author Contributions

RO designed and performed experiments and wrote the manuscript. LP supported the implementation of the model and gave scientific review. FB had the initial idea of using human skin explants for vaccine research and promoted implementation of the model. In addition, he supervised this research study and wrote the manuscript. LG supported the implementation of the model. All the authors approved the manuscript before it was submitted.

## Conflict of Interest Statement

RO is a recipient of GSK fellowship from the Ph.D. program in Biochemistry and Molecular Biology of the University of Siena. FB and LP are employees of GSK group of companies. FB owns patents on *S. aureus* vaccine candidates GSK stocks. LG is an employee of the University of Siena, Italy. The authors have no other relevant affiliations or financial involvement with any organization or entity with a financial interest in or financial conflict with the subject matter or materials discussed in the manuscript apart from those disclosed.
