# Supplementary material for: Deciphering the Pathological Role of Staphylococcal α-Toxin and Panton–Valentine Leukocidin Using a Novel Ex Vivo Human Skin Model
Source: Front Immunol. 2018 May 8;9:951. doi: 10.3389/fimmu.2018.00951 (PMC5953321; doi:10.3389/fimmu.2018.00951)
Supplement: Supplementary file 1 [file Image_1.PDF]

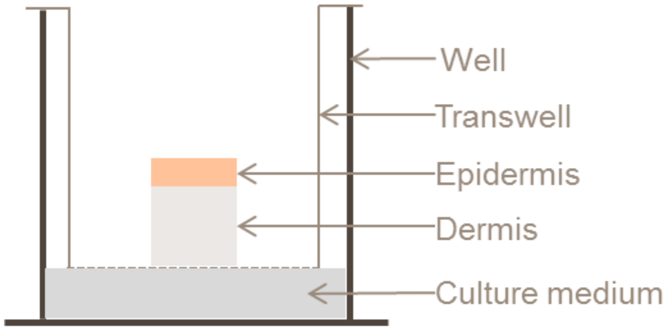

Figure 1. A schematic diagram showing how a human skin biopsy is maintained at air-liquid interface in a transwell filter.
