# Supplementary material for: Deciphering the Pathological Role of Staphylococcal α-Toxin and Panton–Valentine Leukocidin Using a Novel Ex Vivo Human Skin Model
Source: Front Immunol. 2018 May 8;9:951. doi: 10.3389/fimmu.2018.00951 (PMC5953321; doi:10.3389/fimmu.2018.00951)
Supplement: Supplementary file 2 [file Image_2.PDF]

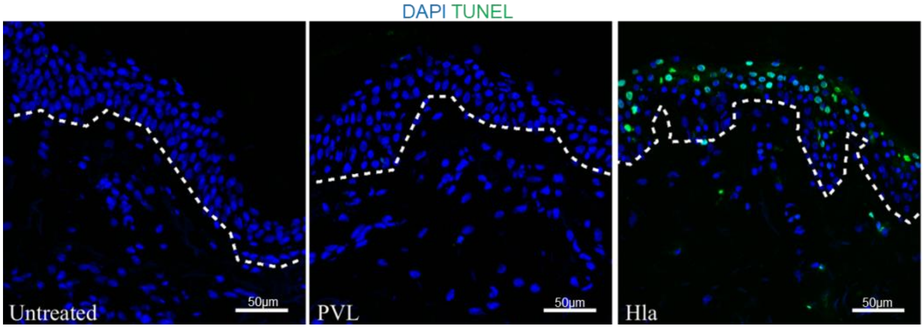

Supplementary Figure 2. Skin sections showing TUNEL positive cells within the epidermis of Hla treated skin 24 hours post-treatment
