# Supplementary material for: Deciphering the Pathological Role of Staphylococcal α-Toxin and Panton–Valentine Leukocidin Using a Novel Ex Vivo Human Skin Model
Source: Front Immunol. 2018 May 8;9:951. doi: 10.3389/fimmu.2018.00951 (PMC5953321; doi:10.3389/fimmu.2018.00951)
Supplement: Supplementary file 3 [file Image_3.PDF]

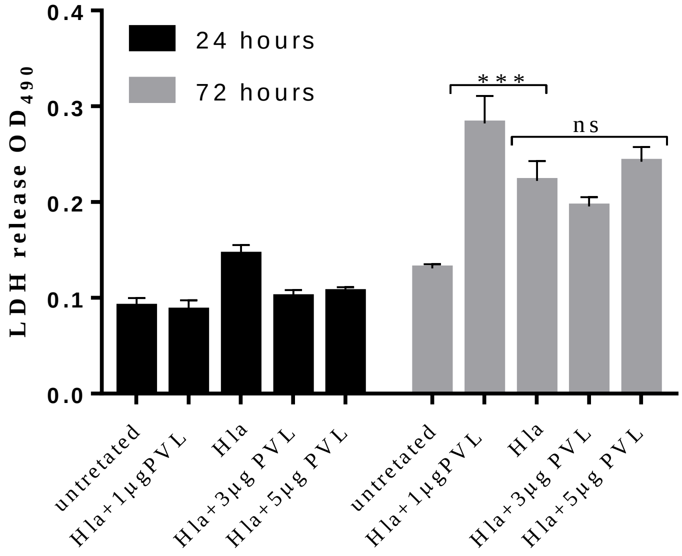

Supplementary Figure 3. LDH release from human skin after treatment with a cocktail of Hla and PVL. Skin explants were treated with 1µg of Hla alone or plus increasing concentration of PVL. Each bar is mean±SD (N=3). Statistically significant differences were determined by two-way ANOVA, with Turkey multiple comparison tests. ***p≤0.0002. Line across bars is comparison with between Hla treatments
