# Supplementary material for: Deciphering the Pathological Role of Staphylococcal α-Toxin and Panton–Valentine Leukocidin Using a Novel Ex Vivo Human Skin Model
Source: Front Immunol. 2018 May 8;9:951. doi: 10.3389/fimmu.2018.00951 (PMC5953321; doi:10.3389/fimmu.2018.00951)
Supplement: Supplementary file 5 [file Image_5.PDF]

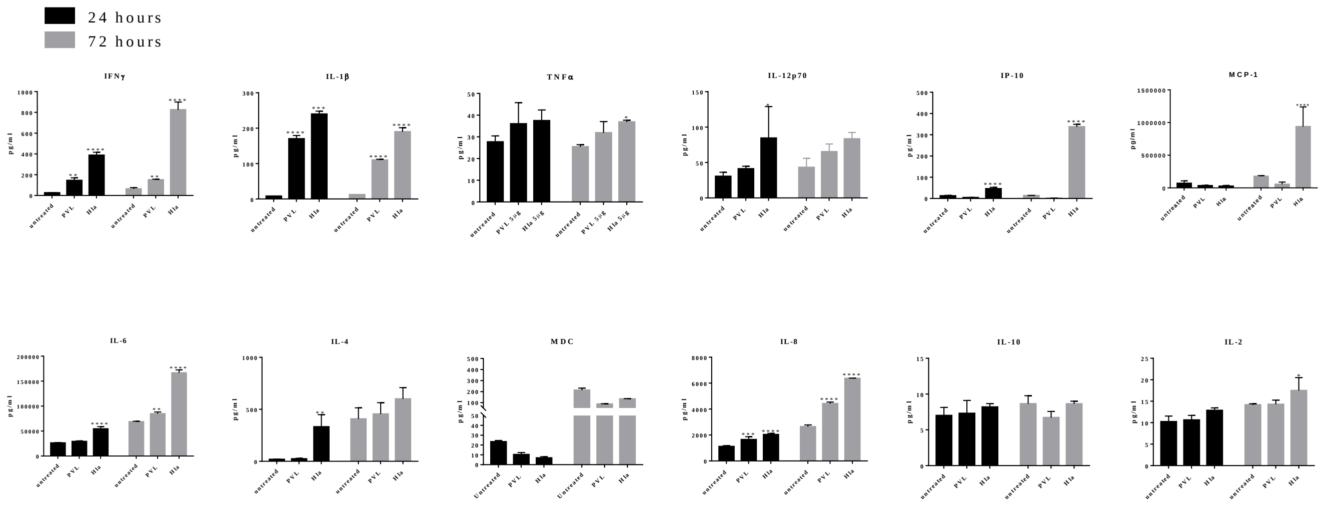

Supplementary Figure 5. Hla and PVL elicit the release of proinflammatory mediators in human skin. Human skin explants were treated with 5µg of Hla or PVL. Each bar is mean ± SD (N=3). Statistically significant differences were determined by two-way ANOVA, with Turkey multiple comparison tests. *$p < 0.05$, **$p \leq 0.001$, ***$p \leq 0.0002$, ****$p < 0.0001$. Asterisks on bars are comparisons between untreated and treated groups..
